# An update on the genetic architecture of hyperuricemia and gout

**DOI:** 10.1186/s13075-015-0609-2

**Published:** 2015-04-10

**Authors:** Tony R Merriman

**Affiliations:** Department of Biochemistry, University of Otago, Box 56, Dunedin, 9054 New Zealand

## Abstract

Genome-wide association studies that scan the genome for common genetic variants associated with phenotype have greatly advanced medical knowledge. Hyperuricemia is no exception, with 28 loci identified. However, genetic control of pathways determining gout in the presence of hyperuricemia is still poorly understood. Two important pathways determining hyperuricemia have been confirmed (renal and gut excretion of uric acid with glycolysis now firmly implicated). Major urate loci are *SLC2A9* and *ABCG2.* Recent studies show that SLC2A9 is involved in renal and gut excretion of uric acid and is implicated in antioxidant defense. Although etiological variants at *SLC2A9* are yet to be identified, it is clear that considerable genetic complexity exists at the *SLC2A9* locus, with multiple statistically independent genetic variants and local epistatic interactions. The positions of implicated genetic variants within or near chromatin regions involved in transcriptional control suggest that this mechanism (rather than structural changes in SLC2A9) is important in regulating the activity of SLC2A9. ABCG2 is involved primarily in extra-renal uric acid under-excretion with the etiological variant influencing expression. At the other 26 loci, probable causal genes can be identified at three (*PDZK1*, *SLC22A11*, and *INHBB*) with strong candidates at a further 10 loci. Confirmation of the causal gene will require a combination of re-sequencing, trans-ancestral mapping, and correlation of genetic association data with expression data. As expected, the urate loci associate with gout, although inconsistent effect sizes for gout require investigation. Finally, there has been no genome-wide association study using clinically ascertained cases to investigate the causes of gout in the presence of hyperuricemia. In such a study, use of asymptomatic hyperurcemic controls would be expected to increase the ability to detect genetic associations with gout.

## Introduction

**Figure 1:**
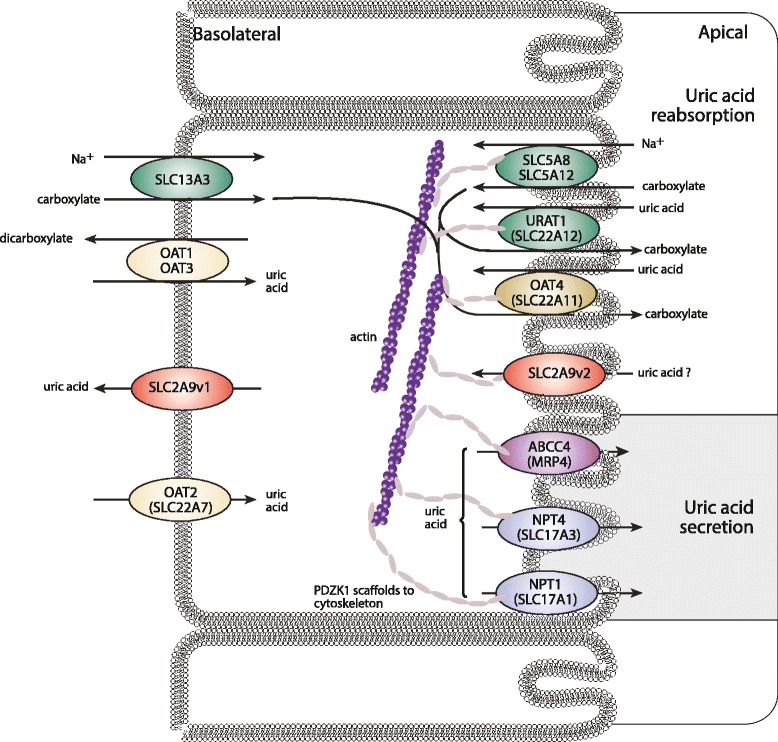
**The uric acid transportasome.** The current understanding of uric acid transport in the proximal renal tubule is presented. Carboxylates accumulate in the tubular cell through sodium-dependent monocarboxylate transporters SLC5A8 and SLC5A12 and through SLC13A3. Uric acid enters the cell in exchange for carboxylate via apical URAT1 and apical OAT4. Apical SLC2A9v2 plays a significant role in uric acid reabsorption within the collecting duct, the reabsorbed uric acid exiting the cell through basolateral SLC2A9v1 in the proximal tubule. For efflux of uric acid into the lumen, MRP4, a voltage-driven organic anion transporter (vOAT1/NPT1), and NPT4 are candidates. OAT1 and OAT3 are known to transport uric acid, although the direction of transport is not clear. PDZK1 is a scaffolding protein involved in assembly of a transporter complex in the apical membrane. Genetic variation in SLC2A9, ABCG2, URAT1, NPT1, OAT4, and PDZK1 is associated with serum urate levels and gout.

Hyperuricemia is necessary but not sufficient for gout. Gout is typically characterized by recurrent self-resolving attacks of acute inflammatory arthritis and occurs in about a quarter of people with elevated serum urate levels (hyperuricemia) [1]. The metatarsal-phalangeal joint of the big toe is most often affected, but gout commonly affects other joints. Two important physiological mechanisms determine hyperuricemia: (a) increased production of the urate in the liver from dietary and endogenous substrates that raise purine levels and (b) reduced renal and gut excretion of uric acid (Figure 1). In the presence of hyperuricemia, factors controlling the formation of monosodium urate (MSU) crystals in synovial fluid and the subsequent innate immune inflammatory response are relatively poorly understood. However, activation of Toll-like receptors and inflammasome-mediated release of the pro-inflammatory cytokine interleukin-1β is known to be a central pathway [2]. Like any other complex phenotype, hyperuricemia and gout result from the interplay between inherited genetic risk variants and environmental exposures [3]. The genetic component will be discussed in this review, and environmental exposures that interact with genetic risk variants will also be considered.

A genome-wide association study (GWAS) scans the genome, in an unbiased fashion using common genetic variants (typically single-nucleotide polymorphisms), for loci causally associated with a particular phenotype. Genes contained within the associated loci are candidates for involvement in causal pathogenic pathways. Köttgen and colleagues [4] reported, in a GWAS of more than 140,000 European individuals, statistically significant associations of 28 separate genetic loci with serum urate levels. This study confirmed the association with urate levels of 10 loci discovered in earlier and smaller GWASs [5-7]. Reviewed elsewhere [8-11], the 10 are dominated by loci containing genes that were either known (*SLC22A11/OAT4*, *SLC22A12/URAT1*, *SLC17A1/NPT*, and *PDKZ1*) or novel (*SLC2A9/GLUT9* and *ABCG2*) renal and gut transporters of uric acid. The *GCKR* (glucokinase regulatory protein) locus implicates production of urate by glycolysis, but the functional relevance of the remaining loci (*SLC16A9/MCT9*, *INHBC*, and *RREB1*) is unclear, although MCT9 may be a renal sodium transporter and has been linked to urate via carnitine metabolism [6]. Predictably most, but not all, of these 10 loci consistently associate with gout in multiple ancestral groups [4,12,13].

The lead associated genetic variants at *SLC2A9* and *ABCG2* collectively explain, depending on sex, 3% to 4% of the variance in urate levels. On average, the urate-raising allele at *SLC2A9* increases serum urate by 0.373 mg/dL (0.022 mmol/L) and the urate-raising allele at *ABCG2* by 0.217 mg/dL (0.013 mmol/L), both of which are clinically significant amounts [4]. *SLC2A9* and *ABCG2* have equivalent effects in men; *SLC2A9* has a stronger effect in women than men and vice versa for *ABCG2* [4]. Sex-specific effects aside, both loci (in particular, *SLC2A9*) exert very strong control on urate levels, when compared with the effect of the other 26 confirmed urate loci that collectively explain a similar proportion of variance. Thus, there is considerable research interest in understanding the molecular basis of urate control by *SLC2A9* and *ABCG2* and their clinical significance.

## Review

### The emerging role of SLC2A9 in metabolism and cancer

A major site of expression of SLC2A9 is the kidney, where it is a voltage-dependent uric acid transporter [14,15]. Genotype-specific expression data are consistent with the possibility that the major causal serum urate-raising variant (that has not yet been genetically pinpointed) increases the expression levels of an SLC2A9 isoform (SLC2A9-S) that has a 28-residue portion missing from the N-terminus [16,17]. This isoform is expressed on the apical (urine) side of the collecting duct, where it presumably increases reuptake of secreted uric acid, whereas the full-length version (SLC2A9-L) is expressed on the basolateral side [14]. Combined with its expression on the basolateral membrane of hepatocytes [18], where urate is generated, the membrane potential would ensure that the SLC2A9-L isoform is responsible for transport of uric acid into the bloodstream [19]. With the caveat that results from *SLC2A9*-inactivation studies in the mouse can only be extrapolated to humans with much caution (given the presence of active urate oxidase (uricase) in mice but not humans), a recent study demonstrates that *SLC2A9* is also an important basolateral uric acid efflux transporter into the gut enterocyte [20]. Interestingly, mice with a gut-specific *SLC2A9* knockout developed a metabolic syndrome-like condition in addition to hyperuricemia [20]. In mice a hepatocyte-specific *SLC2A9* knockout develops severe hyperuricemia, consistent with a role for SLC2A9 in hepatic uptake of uric acid [18]. Because of the presence of uricase, the urate chemical gradient overcomes the membrane potential so that SLC2A9 can transport urate into liver cells. This contrasts with humans, in whom SLC2A9 transports urate out of the hepatocyte [21].

One reason that humans and higher apes have higher urate levels is the postulated function of urate as an antioxidant [22], replacing ascorbic acid as a major endogenous antioxidant in human evolution [23]. Consistent with this hypothesis, intracellular reactive oxygen species (ROS) in cell culture are reduced by physiological levels of urate [24]. Interestingly, oxidative stress induces SLC2A9 transcription and expression in a manner dependent on transcriptional control by the p53 tumor suppressor [24]. Inhibition of SLC2A9 activity by use of small interfering RNAs or the urate-lowering drugs probenecid and benzbromarone increases ROS levels in a urate-dependent manner and increases susceptibility of cancer cells to apoptotic cell death induced by the chemotherapeutic agent cisplatin [24]. Notably, samples from four tumor types (prostate, renal, testis, and andrenal) showed reduced SLC2A9 expression, and survival is better in gastric cancers with higher SLC2A9 expression. Collectively, these data implicate a role for SLC2A9 in countering intracellular ROS by transport of urate (which reduces ROS) and provide support for the controversial hypothesis (based on observational data) linking uric acid to protection from cancer [22,25]. There could be therapeutic potential in inhibiting SLC2A9 in order to chemosensitize cancer cells by increasing ROS levels [24].

**Figure 2:**
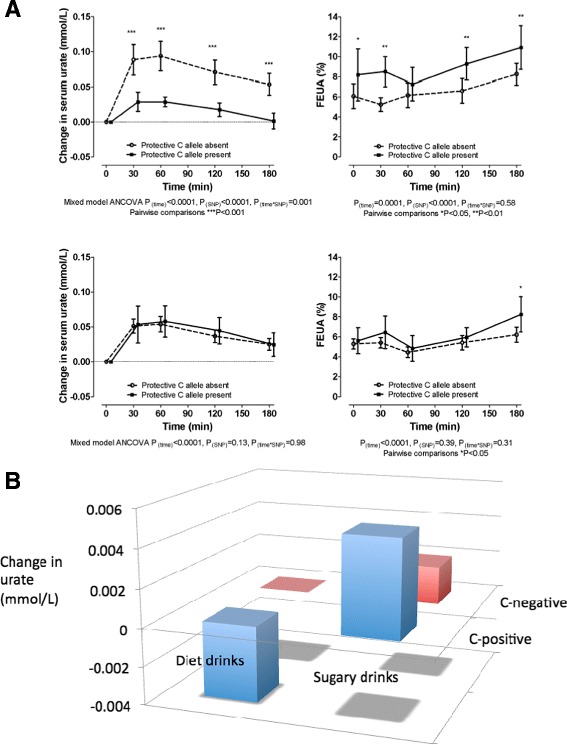
**Interaction between SLC2A9 genotype and sugar exposure.** In both panels, the genetic marker used was *rs11942223* for which the C-positive genotype associates with reduced serum urate. **(A)** Effect of *SLC2A9* genotype on acute response to a fructose load. Change in serum urate is shown on the left, fractional excretion of uric acid (FEUA) on the right. The genotype differences were statistically significant for Europeans (top graphs) but not for Polynesians (bottom graphs). Figure taken from Dalbeth and colleagues [28]. **(B)** The non-additive interaction of sugar-sweetened beverage (SSB) consumption with *SLC2A9* genotype in influence of urate levels in Europeans in the Atherosclerosis Risk in Communities data set [26]. Exposure to artificially (diet) sweetened beverages does not influence the urate-lowering effect of the C-positive genotypes. However, exposure to SSB reverses the urate-lowering effect of the C-positive genotype. The y-axis corresponds to change in urate per consumption category as defined by Batt and colleagues [26]. Data taken from Table 4 of Batt and colleagues [26].

The hepatic metabolism of fructose generates urate through generation of ADP and catabolism through the purine degradation pathway and is one biochemical explanation for the association of sugar-sweetened beverage (SSB) consumption with urate levels and the risk of gout [26,27]. Given that SLC2A9 also transports fructose and glucose [19], it is reasonable to hypothesize that fructose could also directly interfere with renal uric acid transport. Therefore, a recent clinical study examined the *SLC2A9* genotype-dependent acute hyperuricemic response to a fructose load [28]. When a genetic variant (*rs11942223*) largely equivalent to the most strongly associated *SLC2A9* variant in the GWAS by Köttgen and colleagues [4] (Table 1) was used, the urate-lowering allele was associated with an attenuated hyperuricemic response and increased fractional excretion of uric acid (FEUA) in people of European ancestry (Figure 2) [28]. However, despite an appreciable prevalence in participants of New Zealand Polynesian (Maori and Pacific) ancestry (18% versus 32% in Europeans), there was no relationship between positivity for the urate-raising allele and the hyperuricemic and FEUA response to the fructose load, despite prior evidence for association of *rs11942223* with gout in Polynesians [29]. It is possible that there is a Polynesian-specific genetic variant in *SLC2A9* that encodes a functional effect that overrides the genotype-specific FEUA effect seen in European Caucasians.

**Table 1:** **Summary of the 28 genome-wide significant urate loci detected by Köttgen and colleagues** [4]

	**GRAIL gene**	**Effect size (male/female** ^**a**^ **), mg/dL**	**FEUA, Yes/No** ^**b**^	**Association signal**	**Probable causal gene** ^**c**^	**Strongest candidate(s)** ^**d,e**^
Old loci						
Rs1471633	*PDZK1*	0.059	No	Within *PDZK1*	*PDZK1*	*-*
Rs1260326	*GCKR*	0.074 (0.091/0.063)	Yes	Spans >20 genes	-	*GCKR*
Rs12498742	*SLC2A9*	0.373 (0.269/0.460)	Yes	Spans 4 genes	*SLC2A9*	-
Rs2231142	*ABCG2*	0.217 (0.280/0.181)	Yes	Spans 4 genes	*ABCG2*	-
Rs675209	*RREB1*	0.061	Yes	Upstream and within *RREB1*	-	*RREB1*
Rs1165151	*SLC17A3*	0.091	No	Spans 20 genes	-	*SLC17A1-A4*
Rs1171614	*SLC16A9*	0.079	No	Spans 2 genes	-	*-*
Rs2078267	*SLC22A11*	0.073	Yes	Within *SLC22A11*	*SLC22A11*	-
Rs478607	*SLC22A12*	0.047	Yes	Spans 6 genes	-	*SLC22A12*
Rs3741414	*INHBC*	0.072 (0.091/0.057)	No	Spans 7 genes	-	-
New loci						
Rs11264341	*PKLR*	0.050	No	Spans 2 genes	-	-
Rs17050272	*INHBB*	0.035	No	Intergenic	*INHBB*	-
Rs2307384	*ACVR2A*	0.029	No	Spans 3 genes	-	-
Rs6770152	*MUSTN1*	0.044	No	Spans 3 genes	-	-
Rs17632159	*TMEM171*	0.039	No	Intergenic	-	-
Rs729761	*VEGFA*	0.047	No	Intergenic	-	-
Rs1178977	*MLXIPL*	0.047	No	Spans 5 genes	-	*MLXIPL*
Rs10480300	*PRKAG2*	0.035	No	Within *PRKAG2*	-	*PRKAG2*
Rs17786744	*STC1*	0.029	No	Intergenic	-	-
Rs2941484	*HNF4G*	0.044	No	Within *HNF4G*		*HNF4G*
Rs10821905	*ASAH2*	0.057	No	Within *A1CF*		*A1CF*
Rs642803	*LTBP3*	0.036	No	Spans 6 genes	-	-
Rs653178	*PTPN11* ^f^	0.035	No	Spans 3 genes	-	-
Rs1394125	*NRG4*	0.043 (0.061/0.032)	Yes	Spans 4 genes	-	-
Rs6598541	*IGF1R*	0.043	Yes	Within *IGFR1*	-	*IGFR1*
Rs7193778	*NFAT5*	0.046	Yes	Intergenic	-	-
Rs7188445	*MAF*	0.032	No	Intergenic	-	-
Rs7224610	*HLF*	0.042	Yes	Within *HLF*	-	*HLF*
Rs2079742	*C17ORF82*	0.043	No	Downstream and within *BCAS3*	-	-
Rs164009	*PRPSAP1*	0.028	No	Within QRICH2	-	-

^a^Male and female effect sizes are given for loci where there was a significant sex-specific difference. ^b^Fractional excretion of uric acid (FEUA) was tested by Köttgen and colleagues [4] on a considerably smaller subset (n = 6,799), meaning that inadequate power may contribute to lack of association seen at loci of weaker effect. ^c^A probable causal gene either has very strong functional evidence (*SLC2A9* and *ABCG2*) or has strong functional evidence combined with association signal restricted to the gene (*PDZK1* and *SLC22A11*) or has very strong expression single-nucleotide polymorphism (eSNP) evidence (*INHBB*). ^d^A ‘strongest candidate’ is listed when the locus contains a candidate with strong functional evidence (*GCKR*, *SLC17A1-A4*, and *SLC22A12*) or has the association signal tightly restricted to the named gene or has strong eSNP evidence (*MLXIPL*). ^e^RREB1, ras responsive element (zinc-finger) binding protein, has been genetically implicated in type 2 diabetes associated end-stage kidney disease [60]. PRKAG2, protein kinase, AMP-activated, gamma 2 non-catalytic subunit, has been genetically implicated in blood pressure control [61]. HNF4G, hepatocyte nuclear factor 4G, has been genetically implicated in obesity [62]. MLXIPL, carbohydrate element-responsive binding protein, has been identified as a pleiotropic gene for metabolic syndrome and inflammation [63]. ^f^ *PTPN11* is approximately 1 Mb downstream of the association signal and does not harbor any association signal. A1CF, APOBEC1 (APOB mRNA editing enzyme) complementation factor; GRAIL, Gene Relationships Across Implicated Loci; HLF, hepatic leukemia factor; IGFR1, insulin-like growth factor 1 receptor.

An epidemiological observational study also investigated the hypothesis that simple sugar (in the form of SSBs) interacts with the *SLC2A9* genotype in influencing serum urate levels and the risk of gout [26]. Upon exposure to SSB, the normally urate-lowering allele at the *rs11942223* variant has a transmutation of effect and raises urate in response to SSB, an effect not seen with artificially sweetened beverages (Figure 2). A similar pattern was seen in the risk of gout [26]. From the current state of knowledge and given the complexity of urate transport in the renal tubule, it is difficult to propose a plausible mechanism to explain this non-additive interaction. The epidemiological observations also are inconsistent with the increased FEUA in response to an acute fructose load associated with the urate-lowering allele, suggesting that distinct biological mechanisms underlie the observation by Dalbeth and colleagues [28] and the interaction data reported by Batt and colleagues [26]. The effects of chronic exposure to fructose-containing SSB would more likely involve other mechanisms (for example, epigenetic) that influence the expression and activity of SLC2A9.

### Genetic complexity at SLC2A9

**Figure 3:**
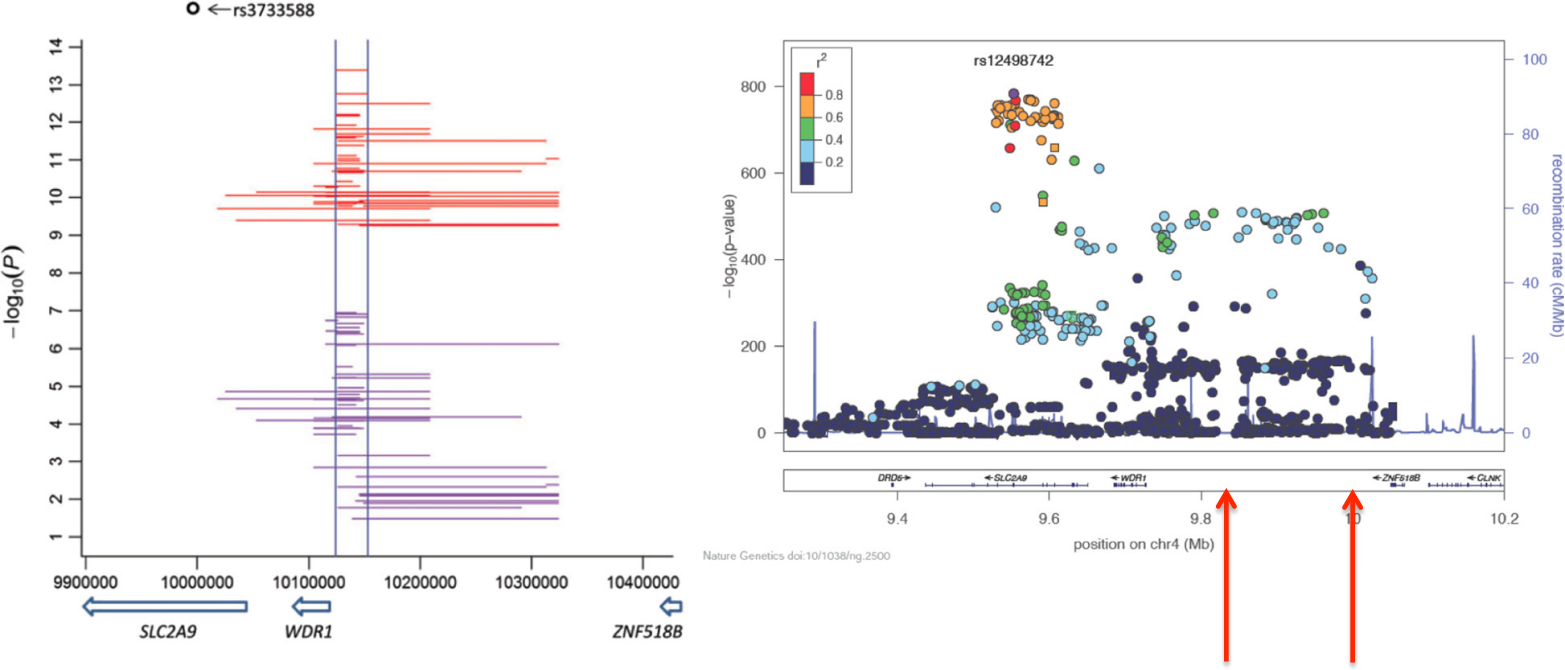
**Genetic complexity of association with urate at SLC2A9.** The left panel, taken from Wei and colleagues [34], illustrates the epistatic SNP-SNP interactions that are present at the *SLC2A9* locus and that concentrate on the indicated 30 kb region. The right panel, taken from Köttgen and colleagues [4], demonstrates the extent of extremely strong association at the *SLC2A9* locus. The approximate positions of the urate-associated copy number variants identified by Scharpf and colleagues [31] are arrowed. The genomic co-ordinates differ between each study because Wei and colleagues [34] used Human Genome Project NCBI build 37.3 and Scharpf and colleagues [31] used NCBI build 36. NCBI, National Center for Biotechnology Information; SNP, single-nucleotide polymorphism.

The urate association signal at the *SLC2A9* locus is extensive with hundreds of genetic variants extremely strongly associated, with the strongest association encompassing a very large region (500 kb) and two genes (*SLC2A9* and *WDR1*) (Figure 3) [4]. *WDR1* encodes a protein involved in disassembly of actin fibers that has been implicated in carditis - not an obvious urate-influencing gene. It is thus difficult to determine whether the genetic effect at *SLC2A9* is caused by a single genetic variant with very strong effect that drives the widespread association owing to extensive intermarker ‘linkage disequilibrium’. This can be studied by ‘conditional analysis’, whereby the association with phenotype of other variants at a locus is tested conditionally on the effect of the strongest associated variant at the locus. Köttgen and colleagues [4] attempted to address this important question and concluded that there was no evidence for multiple independent effects. However, their approach was dictated by a limitation inherent in meta-analyses from many separate studies (n = 48 in their case) in that summary level statistics from each of the studies are combined and it is not possible (for ethical and practical reasons) to combine data from individual participants.

In contrast to the findings of Köttgen and colleagues [4], the possibility of independent effects at *SLC2A9* is supported by two studies. The first is a GWAS of serum urate levels in East Asians [30]. As in Europeans, the strongest genome-wide association with urate was at *SLC2A9*, but with a different single-nucleotide polymorphism (SNP) variant (rs3775948). The most strongly associated variant in the study by Köttgen and colleagues [4] (rs12498742) was not associated in the East Asian GWAS and this was probably because of the rarity of the minor allele (prevalence of approximately 1%). This suggests that there are at least two causal variants controlling urate levels at *SLC2A9*. The second study was a GWAS testing for association of common copy number variation with serum urate in Europeans [31]. This type of variation occurs when chromosomal segments over 1 kb in length deviate from the diploid state, and is a genetic and evolutionary mechanism that can generate significant changes in gene expression from a single mutation event. Examples are the immune CCL3L1 and FCGR3B genes that vary from zero to copy number of greater than four in the human genome. Copy number of these genes is a risk factor for autoimmune disease [32,33]. The only copy number variations associated with urate in the GWAS at a genome-wide level of significance were two separate segments at the *SLC2A9* locus [31]. These variants are 200 kb and 350 kb upstream of *SLC2A9* (Figure 3) and deletion of 12-kb and 7.5-kb segments, respectively, at each copy number variant associates with, respectively, decreased and increased urate levels of approximately 5% in women and approximately 1% in men [31]. Importantly, by conditional analysis, the association of these copy number variants was genetically independent of the previously reported effect at *SLC2A9* [4]. Thus, there is evidence for three independent variants in *SLC2A9* that influence urate levels in Europeans and for a separate variant in East Asians. Although it is not known whether either of the copy number variants is causal or in strong linkage disequilibrium with an unidentified causal variant, at least one is a strong candidate for being causal. The 350-kb upstream variant abuts a DNAse hypersensitivity peak in fetal and adult kidney tissue, suggesting that deletion of the 7.5-kb segment could influence binding of proteins that regulate expression of *SLC2A9* [31].

The study by Wei and colleagues [34] is consistent with the above studies in providing evidence for multiple independent genetic effects at the *SLC2A9* locus; using conditional analysis, they found direct evidence for five independent genetic effects. Furthermore, additional complexity in genetic control of urate levels at *SLC2A9* was revealed*.* In a genome-wide scan in 9,172 individuals of European ancestry for epistasis (non-additive interaction) between genetic variants in influencing urate levels, the only genome-wide significant effects were seen for five SNP pairs at the *SLC2A9* locus, in a 30-kb region upstream of the *WDR1* gene (Figure 3). At least one of these was statistically independent of the aforementioned five independent genetic effects. Collectively, the independent SNPs and the interacting SNP pairs explained 6.0% of the variance in urate levels in the European data set analyzed; this is an exceptionally large effect for a genetic locus regulating a complex phenotype. Evidence for an unusual enrichment of chromatin interactions (mediated by enhancers) was found in both the *WDR1-ZNF518B* and *SLC2A9-WDR1* intergenic regions, which included the interacting SNP pairs. This generates the hypothesis that *SLC2A9* and *WDR1* may be co-transcribed or share transcriptional regulatory machinery. As a final comment, given that SLC2A9 is part of the renal uric acid ‘transportasome’ [35], which contains other genetically regulated uric acid transporters and accessory molecules, it was surprising that epistatic interactions between *SLC2A9* and other loci throughout the genome were not discovered by Wei and colleagues [34]. It will be important to repeat this genome-wide epistasis scan in larger data sets.

### *ABCG2*

Association of the *ABCG2* locus with serum urate was first reported in the GWAS by Dehghan and colleagues [5]. The genetic basis is considerably simpler than that at *SLC2A9*, and the association signal is reported to be driven solely by the rs2231142 (Q141K) variant [36]. This variant is highly likely to be causal [37]. The ABCG2 protein (also known as breast cancer resistance protein) is a multidrug transport protein transporting a wide range of molecules, including chemotherapeutic agents. It is a secretory uric acid transporter in the proximal tubule and the gut [36,38]. Interestingly, the urate-increasing allele at rs2231142 (141 K) is associated with increased urinary uric acid output [38,39]. In mice, an *Abcg2* knockout also showed increased renal but decreased gut uric acid excretion [38]. This allele was also associated with a reduced increase in serum urate and glucose in response to a fructose load [39]. Collectively, these results show that the urate-increasing allele at ABCG2 does not act directly via direct effects on renal uric acid transport but through increased gut excretion. Histone deacetylase inhibitors are able to correct the ABCG2 141 K urate-increasing ‘defect’ [37]. ABCG2 Q141K may also interact with extra-renal metabolic pathways to regulate serum urate (for example, via an influence on hepatic conversion of fructose to glucose) [39]. Ichida and colleagues [38] propose that ABCG2 defines one of three pathways contributing to hyperuricemia, namely extra-renal uric acid under-excretion, the other two being genuine urate over-production and renal uric acid under-excretion.

### The study by Köttgen and colleagues

The large GWAS by Köttgen and colleagues [4] reported 18 new loci with a weaker effect on urate levels than the previously identified 10; the new 18 explained a further 1.8% of variance in urate levels compared with 5.2% for the 10 previously known loci. Notably, none of the new loci contained genes encoding known uric acid transporters, although an association with almost genome-wide significance was detected in the *SLC2A7* locus (encoding the organic anion transporter 2) in a candidate gene secondary analysis. Summarized in Table 1, the study by Köttgen and colleagues contains a treasure trove of information on the control of urate levels.

Candidate genes at each locus were identified by Köttgen and colleagues by using Gene Relationships Across Implicated Loci (GRAIL) [40], a bioinformatic approach that looks for commonalities between associated SNPs, the literature, and published GWASs. The GRAIL genes were mapped into two broad pathways: glycolysis and inhibins/activins. The relevance of the glycolysis genes to urate likely reflects hepatic production of urate (from sugar and alcohol) via increased generation of glucose-6-phosphate that flows through the pentose-phosphate pathway generating ribose-5-phosphate, a precursor of purine synthesis. Generation of lactic acid from anaerobic glycolysis could also interfere with renal uric acid excretion. This possibility is consistent with the strong association of the *GCKR* locus with fractional excretion of uric acid (the GCKR protein inhibits glucokinase that produces glucose-6-phosphate) [4]. Köttgen and colleagues noted that the associations with loci containing genes involved in glucose homeostasis fit with the observation that drugs that decrease insulin resistance (for example, metformin) also tend to decrease serum urate levels, indicating possible new approaches for management of urate levels. The relevance of the inhibins/activins is not clear; Köttgen and colleagues [4] suggested processes such as energy balance, insulin release, apoptosis, inflammation, and sex hormone regulation.

**Figure 4:**
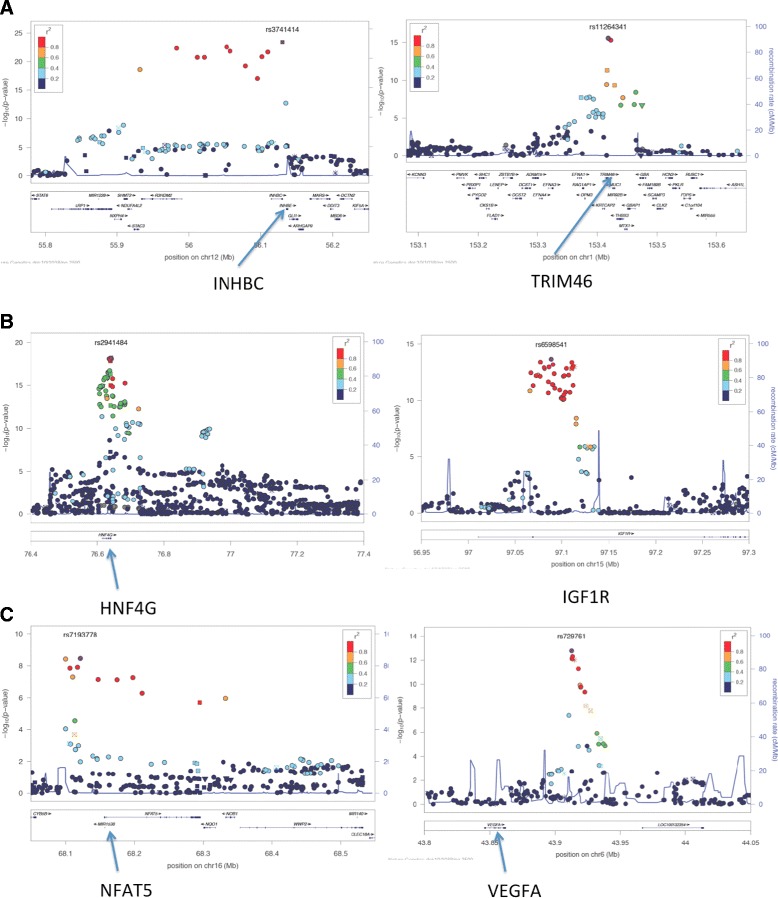
**LocusZoom pictures of regional association in Europeans in the study by Köttgen and colleagues [** 4 **].** The top associated single-nucleotide polymorphism (SNP) is labeled, and other associated SNPs are colored according to strength of linkage disequilibrium (red = high; purple = very low). –log_10_P is on the left-hand y-axis. **(A)** Illustrating multiple genes underlying a serum urate association signal at the INHBC and TRIM46 loci. **(B)** Examples of association signals that define a single causal gene of high-prior probability. **(C)** Examples of intergenic association signals.

There is one very important caveat in interpreting the GWAS findings: the considerable majority of the GRAIL-identified genes cannot be assumed as causal. Extensive linkage disequilibrium (intermarker correlation) results in association signals extending for some distance across many loci. This means that multiple candidate genes can exist (see examples in Figure 4). To identify the causal gene at each locus will require further genetic research, beginning with resequencing of candidate genes in each locus with the causal gene predicted to have a larger burden of rare functional variants in extreme hyperuricemia. This approach can be complemented by trans-ancestral mapping with the most likely common causal variant (that is, the effect identified by Köttgen and colleagues) predicted to be most strongly associated with urate levels (and gout) between diverse ancestral groups. Alongside this approach, identification of ancient recombinant haplotypes that differ between ancestral groups can aid in fine-mapping. A third approach that was employed by Köttgen and colleagues [4] to identify likely candidate genes is underpinned by the hypothesis that the causal variant is an ‘eSNP’ (expression SNP) that influences the expression of the causal gene at the locus. This is a strong hypothesis given that approximately 70% of genetic variants for common phenotypes identified by GWASs map to regulatory regions of the genome [41]. The authors correlated the significant urate-associated SNPs with expression of genes in various tissues from publically available databases. The tissues included various white blood cells, adipose, various neural cells, fibroblasts, osteoblasts, and liver, although no renal tissue or cell line was analyzed. Of the total 28 genome-wide significant loci, eight showed strong (*P* < 1 × 10^−4^) evidence for association with multiple expression probes. Notable in this analysis was clear evidence that the intergenic association signal at the *INHBB* locus (Figure 4) was associated with expression of INHBB in the liver. This provides evidence that *INHBB* is the causal gene at this locus. At *ABCG2*, the rs2231142 variant (Q141K), which is highly likely to be a causal variant at this locus [36], was associated with *ABCG2* expression in the liver [4]. This is consistent with functional evidence that the urate-increasing allele (141 K) reduces ABCG2 protein expression levels [37] and with the hypothesis that ABCG2 (known to transport uric acid [36]) operates in extra-renal pathways to influence urate levels [39]. There was association with expression of both *BAZ1B* and *MLXIPL* in adipose tissue at the *BAZ1B* locus. This may reflect co-ordinated expression of closely linked genes but is consistent with the role of *MLXIPL* (which encodes the glucose-responsive transcription factor ChREBP) in transcriptional activation of glycolytic genes. Interpretation of results at the remaining five loci (*TRIM46*, *GCKR*, *SFMBT1*, *SLC17A1*, and *ATXN2*) is less obvious. For example, there was strong association with multiple expression probes in neural expression data sets with the *CUX2* gene at the *ATXN2* locus. The eSNP approach does need to be reapplied to the 28 urate loci by using a wider range of tissue expression data sets that include renal tissue and gut enterocytes from different developmental stages.

There are some loci where the causal gene appears obvious (*HLF*, *HNF4G*, *IGF1R*, and *PRKAG2*) where the associated signal is tightly restricted within a single gene (Table 1 and Figure 4). However, other approaches (genetic and functional) are required to confirm these as the causal genes. The FTO locus in weight control is a salutary example. Given the tight restriction of the association signal to the FTO gene, it has been widely assumed that the causal effect owes to the FTO protein. However, it has now been shown that the weight-associated variants in FTO interact with the promoter of the neighboring *IRX3* gene [42], suggesting that *IRX3* may mediate the effect of the association signal at *FTO.* Some signals are tightly restricted to an intergenic segment (*INHBB*, *MAF*, and *VEGFA*), indicating that urate control is probably enhancer-mediated by control of expression of the causal gene. These association signals illustrate that the considerable majority of common genetic variants associated with human phenotypes map to functionally important regions of the genome that regulate gene expression [41]. As illustrated by INHBB [4], the eSNP approach will be particularly useful in these situations.

### Genetic contribution in different ancestral groups

Population groups such as Taiwanese Aborigines and Polynesians (primarily Samoan, Tonga, Niuean, Tokelauan, and Cook Island and New Zealand Maori) have inherently higher serum urate levels, as evidenced by mid-20th century epidemiological studies and prehistoric evidence for gout [43]. The contemporary populations have gout prevalences of more than double those of other population groups (Europeans, for example [44]). This suggests that an increased prevalence of urate-raising genetic variants, some of which may be unique, contributes to urate-raising and risk of gout. This hypothesis has generally been difficult to assess, with only urate-raising variants discovered in Europeans examined thus far. There is, however, some indication that prevalences of urate-raising alleles are higher and effect sizes are stronger in Taiwanese Aborigines and Polynesians [45,46]. To better evaluate the possible contribution of population-specific genetic variants, known and candidate urate loci need to be resequenced in Taiwanese Aborigines and Polynesians.

### The urate-associated genetic variants as tools for Mendelian randomization studies

An important biomedical question is whether hyperuricemia and gout are causal of associated metabolic conditions such as hypertension and heart and kidney disease. Observational studies that account for measured confounders suggest that hyperuricemia is causal. However, these studies, no matter how well designed, cannot remove all sources of confounding. Because genetic variants associated with phenotype are biological exposures present since conception, the biological processes they influence can be regarded as causal of phenotype. This tenet provides the basis for the Mendelian randomization genetic technique that is increasingly being applied to understand biological cause-effect relationships and that removes confounding as a fundamental issue in disentangling cause-effect relationships. This technique can be likened to a randomized clinical trial, whereby individuals are randomly assigned by nature to separate exposure (allele that raises biological exposure of interest) and control (other allele) groups at gamete formation and conception and followed for disease outcome. Using urate-associated genetic variants, particularly those within the *SLC2A9* locus, as surrogates for the exposure (urate) Mendelian randomization has provided evidence that urate is not causal for ischemic heart disease, metabolic syndrome, reduced renal function, or increased triglyceride levels [47-50].

### The missing heritability in urate control: common variants and gene-environment interaction

Heritability is defined as the proportion of phenotypic variability that is explained by inherited genetic variants. In humans, it is usually calculated from twin studies that compare phenotypic concordance between mono- and di-zygotic twin pairs. It includes all genetic effects, including epistasis (non-additive genetic interactions) and non-additive interactions with environmental exposures (GxE). Heritability of urate levels is estimated to be approximately 60% [51]. Typical of the situation for other complex phenotypes, the proportion of variance in urate levels explained by common genetic variants detected by GWAS is low (7.0%) [4], accounting for only a small proportion of the genetic component. This problem has been termed the ‘missing heritability’ [52], with the explanation(s) for this phenomenon unresolved.

In GWAS data sets, calculations to estimate the contributions of SNPs to heritability use cross-sectional case–control data. These yield the ‘narrow-sense’ heritability (*h*^*2*^), in which only the heritability from the average effect of genetic variants acting independently (additively) is estimated and contributions from epistasis and non-additive gene-environment (GxE) interactions are ignored. There are various theories to explain the missing heritability: (a) that common causal variants of weak effect go undetected in GWASs, (b) that undetected rare variants with larger effect sizes contribute, (c) that the heritability estimates of known genetic variants that are derived from the narrow-sense models are under-estimated owing to the unaccounted contribution of epistasis between loci, and (d) similarly that narrow-sense heritability estimates are under-estimated because of the unaccounted for contribution of non-additive GxE effects. In control of urate, there is evidence to support possibility (a); 27% to 41% (depending on the data set) of heritability is explained when all common SNPs and not just the statistically significant SNPs are considered [4]. It will be possible to address possibility (b) when all genetic variation can be evaluated from large whole-genome sequence data sets. Improvement in analytical approaches and computational power will allow testing of (c) from current GWAS data. Regarding (d), there is evidence that non-additive GxE interactions will explain an under-appreciated proportion of the missing heritability in urate. In addition to the *SLC2A9-*SSB interaction discussed previously [26], there is non-additive interaction between alcohol exposure and the lipoprotein receptor-related protein 2 gene (*LRP2*) (*rs2544390*) in the risk of gout in Polynesian populations, where the protective effect of the T-positive genotype is negated by exposure to alcohol [53]. Finally, a non-additive interaction between diuretic use and genotype at each of *SLC2A9* and *SLC22A11* in the risk of gout in hypertensive people has been reported in the Atherosclerosis Risk in Communities study [54]. These findings, subject to wider replication and support from interventional studies, also raise the possibility of personalized approaches to the management of hyperuricemia.

### Association of the urate loci with gout

As expected, most of the urate loci are also risk factors for gout [4,12,13], with only four loci (*INHBB*, *HNF4G*, *UBE2Q2*, and *BCAS3*) yet to be formally associated with gout at a nominal level of significance. Use of larger gout case sample sets should enable this to be done. The urate-raising allele in Europeans is associated with increased risk of gout in the considerable majority of circumstances, including in Polynesians [12]. Exceptions to this are *PRKAG2* and *HLF* where the European urate-raising allele protects from gout in Polynesians [12]. This observation could be useful in fine-mapping the causal variant at each locus under the hypothesis that the associated SNPs (*rs10480300* and *rs7224610*) are not causal and that there is a recombinant haplotype differentiating gout risk at these loci. The causal variant can be expected to map to surrounding DNA where the same allele of genetic variants would consistently associate with risk of gout in both ancestral groups.

While correlation between increased effect size on urate and effect size on gout is seen [4], it is logical to expect that genetic variants with a similar effect on serum urate should have a similar effect on the risk of gout. However, this is not necessarily the case. The risk alleles of *GCKR*, *SLC16A9*, *SLC22A11*, and *INHBC* are associated with an average increase in serum urate of 0.004 mmol/L [4]. Of these loci, *GCKR* has an effect size that is consistently higher in gout; *GCKR* is associated with gout in European, Chinese, Japanese, and Polynesian sample sets (odds ratio (OR) = 1.3 to 1.5 in sample sets where gout is clinically ascertained) [4,12,13,55]. *INHBC* is also consistently associated in European and Polynesian though with a lower OR of approximately 1.15 [4,12]. In contrast, *SLC22A11* is not consistently associated with gout, and the evidence for association reported by Köttgen and colleagues [4] in Europeans (OR = 1.14) has not been replicated elsewhere (OR = 0.98) [45]. The even weaker evidence for association of *SLC16A9* with gout in Köttgen and colleagues [4] (OR = 1.10, *P* = 0.017) was also not replicated elsewhere (OR = 1.01) [12]. So there are clearly inconsistent effects on association with gout among the four loci with very similar effects on serum urate. These observations may result from a lack of independence between molecular pathways of serum urate control and clinical presentation of gout in the presence of hyperuricemia (that is, pleiotropic effects of the urate-associated loci) or from confounding of serum urate and risk of gout effect sizes by unmeasured or unaccounted for environmental exposures (for example, as seen at *SLC2A9*) or from both. In future clinical and epidemiological studies, it will be important to investigate why loci such as *SLC16A9* and *SLC22A11* inconsistently associate with gout.

### Genetics of gout in the presence of hyperuricemia

The heritability of gout is unclear, and the only twin study reported a wide 95% confidence interval (0% to 58.1%) [51]. Despite this uncertainty, it is reasonable to expect that genetic variants control the development of gout in the presence of hyperuricemia; although hyperuricemia is necessary for gout, it is not sufficient as not all hyperuricemic people develop gout. The strongest candidate genes are those influencing the innate immune recognition of and response to MSU crystals, although genes involved in MSU crystal formation are possible. However, there is only one replicated association of an immune gene with gout: an SNP within the candidate *TLR4* innate immune gene is associated with gout in Chinese (OR = 1.42, *P* < 1 × 10^−4^) [56]. This association was not evident in Europeans when unstratified controls are used (OR = 1.26, *P* = 0.10). Importantly, however, the effect size increases considerably and the association is statistically significant when asymptomatic hyperuricemic controls are used (OR = 1.63, *P* = 0.009) [57].

The largest gout GWAS published to date used 3,000 European cases nested within the cohorts used in the urate GWAS by Köttgen and colleagues [4]. The gout GWAS yielded disappointing results; only *SLC2A9* and *ABCG2* were associated at a genome-wide level of significance. A major reason for this is the phenotyping where cases were ascertained by self-report or the use of allopurinol (which is also used in asymptomatic hyperuricemia) or both, resulting in ‘case’ sample sets that will include participants without gout. The *SLC17A1* locus has the third strongest effect on serum urate [4] and has been associated with gout in candidate gene studies in Japanese, European, and Polynesian sample sets ascertained by clinical assessment, where the OR was consistently approximately 1.5 [58,59]. Notably, the effect size for *SLC17A1* in the aforementioned gout GWAS was considerably weaker at an OR of 1.16 [4]. Although there was significant association when the locus was specifically tested (*P* = 0.01), the weaker effect meant that the signal was hidden in the statistical noise inherent in a GWAS. Thus, there is a need for a gout GWAS in clinically ascertained sample sets in order to identify non-serum urate genetic risk factors for gout (for example, TLR4) which are likely to have weak effects (OR <1.4). Ideally, such a GWAS would use people with asymptomatic hyperuricemia as controls, who would be expected to have inherited genetic variants protecting from development of gout in the presence of hyperuricemia.

## Abbreviations

eSNP: Expression single-nucleotide polymorphism
FEUA: Fractional excretion of uric acid
GRAIL: Gene Relationships Across Implicated Loci
GxE: Gene-environment
GWAS: Genome-wide association study
MSU: Monosodium urate
OR: Odds ratio
ROS: Reactive oxygen species
SNP: Single-nucleotide polymorphism
SSB: Sugar-sweetened beverage
